# Nucleotide precursors prevent folic acid-resistant neural tube defects in the mouse

**DOI:** 10.1093/brain/awt209

**Published:** 2013-08-09

**Authors:** Kit-Yi Leung, Sandra C.P. De Castro, Dawn Savery, Andrew J. Copp, Nicholas D.E. Greene

**Affiliations:** Neural Development Unit and Birth Defects Research Centre, Institute of Child Health, University College London, UK

**Keywords:** neural tube defects, spina bifida, embryo, nucleotides, *curly tail*

## Abstract

Closure of the neural tube during embryogenesis is a crucial step in development of the central nervous system. Failure of this process results in neural tube defects, including spina bifida and anencephaly, which are among the most common birth defects worldwide. Maternal use of folic acid supplements reduces risk of neural tube defects but a proportion of cases are not preventable. Folic acid is thought to act through folate one-carbon metabolism, which transfers one-carbon units for methylation reactions and nucleotide biosynthesis. Hence suboptimal performance of the intervening reactions could limit the efficacy of folic acid. We hypothesized that direct supplementation with nucleotides, downstream of folate metabolism, has the potential to support neural tube closure. Therefore, in a mouse model that exhibits folic acid-resistant neural tube defects, we tested the effect of specific combinations of pyrimidine and purine nucleotide precursors and observed a significant protective effect. Labelling in whole embryo culture showed that nucleotides are taken up by the neurulating embryo and incorporated into genomic DNA. Furthermore, the mitotic index was elevated in neural folds and hindgut of treated embryos, consistent with a proposed mechanism of neural tube defect prevention through stimulation of cellular proliferation. These findings may provide an impetus for future investigations of supplemental nucleotides as a means to prevent a greater proportion of human neural tube defects than can be achieved by folic acid alone.

## Introduction

Closure of the neural tube is an essential step in development of the CNS (Copp and Greene, 2010). Failure results in neural tube defects, including spina bifida and anencephaly, severe congenital malformations affecting 0.5–2 per 1000 pregnancies worldwide (Mitchell, 2005). Owing to their occurrence during early development and associated difficulties of diagnosis and remediation, primary prevention is the optimal therapeutic approach. Prevention of neural tube defects became a realistic proposition following the finding that maternal supplementation with folic acid significantly reduces the rate of recurrence, following an affected pregnancy (Smithells *et al.*, 1980; Wald *et al.*, 1991). Consequently, folic acid supplements are recommended before pregnancy. Implementation of food fortification has been associated with reduced neural tube defect prevalence in several countries (Mills and Signore, 2004; Ricks *et al.*, 2012). Nevertheless, data from clinical trials, epidemiological studies and reported occurrences of sequential affected pregnancies in some individuals despite use of folic acid all show that a proportion of neural tube defects are unresponsive to folic acid (Wald *et al.*,1991; Blom *et al.*, 2006; Mosley *et al.*, 2009). Moreover, in the fortified US population, there is no apparent benefit of additional supplement usage, suggesting that simply increasing dosage may not achieve significant further protection (Mosley *et al.*, 2009).

Overall, the relationship between folate status and neural tube defect susceptibility is not well understood, but genetically-determined abnormalities of folate one-carbon metabolism (FOCM) appear to be risk factors. For example, variants in *MTHFR*, *MTHFD1L* and genes encoding the glycine cleavage system (*GLDC* and *AMT*) have been associated with predisposition to neural tube defects (Blom *et al.*, 2006; Greene *et al.*, 2009; Parle-McDermott *et al.*, 2009; Narisawa *et al.*, 2012). Furthermore, a proportion of neural tube defect-derived cell lines exhibit apparent abnormalities of FOCM, indicated by diminished thymidylate biosynthesis (Dunlevy *et al.*, 2007). In mice, loss of function models of *Amt* and *Mthfd1L* exhibit neural tube defects, as do *Shmt* null embryos when under conditions of maternal dietary folate-deficiency (Beaudin *et al.*, 2011; Narisawa *et al.*, 2012; Momb *et al.*, 2013).

FOCM comprises a complex network of reactions with several outputs, including provision of one-carbon units for methylation reactions and nucleotide biosynthesis (Blom *et al.*, 2006; Stover, 2009). One possibility is that some neural tube defects may be resistant to folic acid prevention owing to suboptimal activity of the intervening reactions required to mediate its biological activity. It has been proposed that the protective effect of folic acid may be mediated through an epigenetic mechanism, involving enhanced DNA and/or histone methylation. An alternative, although not exclusive, hypothesis envisages the key requirement for FOCM in neural tube closure to involve provision of nucleotides to sustain rapid cellular proliferation in the neurulating embryo. This raised the question of whether direct supplementation with nucleotides could influence neural tube closure.

We tested the potential effect of nucleotide supplementation in the *curly tail* (*ct*) mouse, a well-established model for human neural tube defects (Van Straaten and Copp, 2001). The major *curly tail* gene corresponds to a hypomorphic allele of the transcription factor *Grhl3*, resulting in diminished messenger RNA expression, and neural tube defects can be rescued by transgenic overexpression of *Grhl3* (Gustavsson *et al.*, 2007). The frequency of spina bifida and exencephaly in the *curly tail* strain is influenced by environmental factors and genetic background, a recently identified modifier gene being lamin B1 (Neumann *et al.*, 1994; De Castro *et al.*, 2012). Importantly, there is no protective effect of folic acid and the *curly tail* strain therefore provides a model for resistant neural tube defects in which to test alternative treatments.

## Materials and methods

### Mice and supplementation

*Curly tail* mice were maintained as a closed random-bred colony (Van Straaten and Copp, 2001). Experimental litters were generated by overnight mating, with the day of finding a copulation plug designated embryonic Day 0.5. Maternal supplementation was performed daily from Days 7.5–10.5. Treatment comprised intraperitoneal injection with stock solutions (in sterile water), containing each reagent at a final concentration of 2 mg/ml, to a dosage of 20 mg/kg. Doses were based on previous studies of thymidine treatment in neurulation-stage embryos (Wlodarczyk *et al.*, 2006; Burren *et al.*, 2008). Treatments were: control (water only); thymidine + adenine; thymidine + hypoxanthine; thymidine + GMP (guanosine monophosphate); thymidine only; and adenine only (all reagents from Sigma). GMP was used instead of guanine owing to solubility considerations in preparing solutions for injection. Animal studies were carried out under regulations of the Animals (Scientific Procedures) Act 1986 of the UK Government, and in according with guidance issued by the Medical Research Council, UK in ‘Responsibility in the Use of Animals for Medical Research’ (1993).

### Collection of embryos

Litters were dissected from the uterus at Days 11.5–13.5, in Dulbecco’s modified Eagle’s medium containing 10% foetal calf serum and assessed for presence of neural tube defects under a light microscope. Any resorptions were recorded. Crown-rump length was measured using an eyepiece graticule.

### Incorporation of nucleotides *in utero* and in embryo culture

Embryos were explanted at Day 9.5 and cultured for 24 h in rat serum as described previously (Cockroft 1990; Greene *et al.*, 2003), in the presence of ^3^H-thymidine, ^3^H-hypoxanthine or ^3^H-adenine (at 1 or 2 µCi/ml). Genomic DNA was isolated and incorporation of ^3^H determined by scintillation counting as described previously (Dunlevy *et al.*, 2007). DNA concentration was determined by NanoDrop.

### Proliferation analysis

Immunohistochemistry for phosphohistone H3 was performed on transverse 7-μm sections at the axial level of the closing neural folds (5–7 sections per embryo) in embryos at Day 10.5 (28–31 somites) as described previously (Gustavsson *et al.*, 2007). Primary and secondary antibodies were anti-phosphohistone H3 (1:250, Millipore) and Alexa Fluor® 488-conjugated anti-rabbit (1:500, Invitrogen). For nuclear staining, cells were incubated with Hoechst (1:2000 in PBS). Fluorescent images were collected on an Axiophot microscope (Zeiss) with a DC500 camera (Leica), using FireCam software (Leica). Images were analysed using the Cell Counter plugin of ImageJ software (U.S. National Institutes of Health). Cells in mitosis were scored by visual inspection of pH3-positive cells.

### Statistical analysis

Statistical analysis was carried out using SigmaStat (version 3.5; Systat Software Inc). Proportions were compared by *z*-test. Comparisons of mean values were made by *t*-test or by one-way ANOVA with pairwise analysis by Holm-Sidak test.

## Results

**Figure 1:**
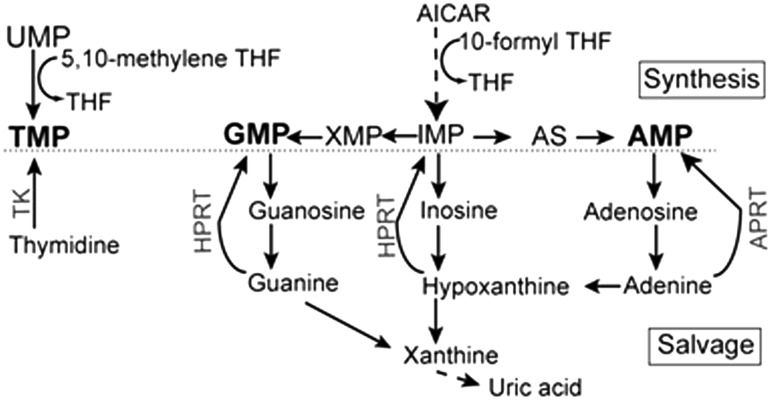
Summary of nucleotide biosynthesis and salvage pathways. Folates, based on a tetrahydrofolate (THF) backbone, contribute one-carbon units for *de novo* synthesis of (**A**) pyrimidine and (**B**) purine nucleotide precursors (in bold; TMP, AMP and GMP). A salvage reaction mediated by thymidine kinase (TK) also allows usage of thymidine as the nucleoside precursor of thymidine monophosphate, while the purine bases adenine and hypoxanthine can be salvaged by the action of adenine phosphoribosyltransferase (APRT) and hypoxanthine phosphoribosyltransferase (HPRT), respectively. AMP = adenosine monophosphate; TMP = thymidine monophosphate.

FOCM supplies one-carbon units in pyrimidine production during biosynthesis of thymidine monophosphate (Fig. 1A) and at two steps in purine biosynthesis leading to production of inosine monophosphate, the precursor of both adenosine monophosphate and GMP (Fig. 1B). In order to provide precursors for these nucleotides we supplemented *curly tail* mice with thymidine (nucleoside precursor of thymidine monophosphate) in combination with adenine or hypoxanthine (purine base precursors of adenosine monophosphate and inosine monophosphate) or with GMP (nucleotide). We hypothesized that provision of precursors of both pyrimidines and purines might produce a greater biological effect than each individually.

**Figure 2:**
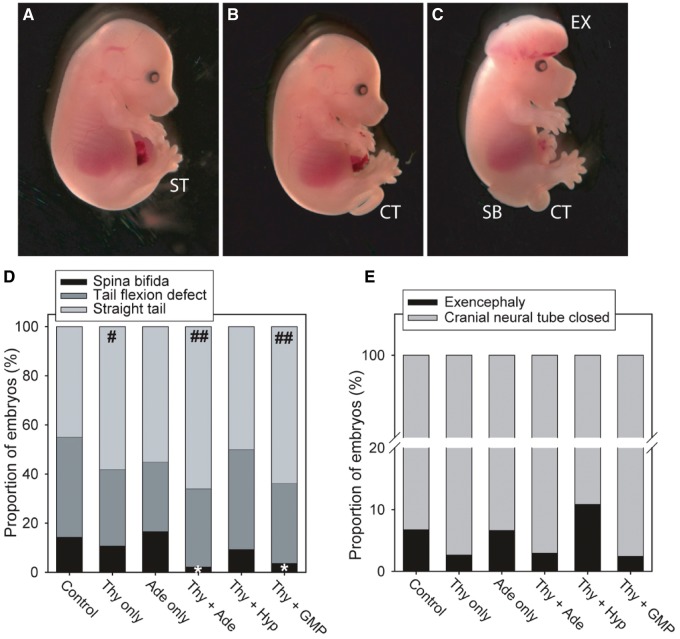
Nucleotide supplementation prevents neural tube defects. The range of phenotypes among control and treated embryos includes (**A**) unaffected with straight tail (ST), (**B**) tail flexion defect/*curly tail* (*CT*) and (**C**) spina bifida (SB) with *curly tail*. Exencephaly (EX) can occur in isolation or with *curly tail* or spina bifida. Representative untreated *curly tail* embryos at embryonic Day 15.5 are shown. The frequency of spina bifida (**D**) was significantly lower among offspring of mice supplemented with thymidine + adenine (*n* = 88 embryos) or thymidine + GMP (*n* = 80) than among controls (*n* = 187 embryos; *P < 0.01; *z*-test). There was a trend towards reduced spina bifida frequency among embryos exposed to thymidine (Thy) only (*n* = 74) or thymidine + hypoxanthine (*n* = 64) but this was non-significant, whereas adenine (Ade) only (*n* = 60) had no effect. There was a significantly higher frequency of straight tails among offspring of mice supplemented with thymidine, thymidine + adenine or thymidine + GMP than among control litters (^#^*P* < 0.01, ^##^*P* < 0.001; *z*-test), correlating with reduced rates of spina bifida in these groups. (**E**) The frequency of exencephaly (cranial neural tube defects) was 6.8% among embryos from control litters. Among embryos exposed to thymidine only, thymidine + adenine or thymidine + GMP there was a trend towards reduced frequency of exencephaly (2.7%, 3.0% and 2.5%, respectively). Thy = thymidine; Ade = adenine; Hyp = hypoxanthine.

Embryos were removed from pregnant females at Days 11.5–13.5. They were scored for the outcome of low spinal neurulation as: normal (straight tail; Fig. 2A), tail flexion defect (curled tail; Fig. 2B), or open spina bifida that was always associated with a tail flexion defect (Fig. 2C). Tail flexion defects arise when closure of the neural tube is delayed but does ultimately occur (Copp, 1985). Individual administration of thymidine or adenine did not significantly alter spina bifida incidence (Fig. 2D). In contrast, combined supplementation produced a striking protective effect, with 85% lower frequency of spina bifida among embryos treated with thymidine + adenine than controls (Fig. 2D). We also observed a significant protective effect of thymidine + GMP, but not thymidine + hypoxanthine (Fig. 2D). In agreement with these findings, analysis of the data on a litter-by-litter basis showed that the mean proportion of embryos with spina bifida per litter was significantly lower for mice treated with thymidine + adenine and thymidine + GMP than for controls (Supplementary Fig. 1A).

In addition to open spina bifida, we analysed the frequency of tail flexion defects, which arise when neural tube closure is delayed, and straight tails, which occur among embryos in which spinal neurulation is apparently normal. Correlating with the data for spina bifida, the frequency of straight-tailed embryos, unaffected by either spina bifida or tail flexion defects, was significantly higher in the thymidine + adenine and thymidine + GMP groups (Fig. 2D and Supplementary Fig. 1B). There was also a higher frequency of unaffected (straight-tailed) embryos among offspring of mice treated with thymidine only (Fig. 2A), although this was not statistically significant in the litter-by-litter analysis (Supplementary Fig. 2B).

Cranial neurulation may also be enhanced by nucleotide supplementation, as exencephaly (Fig. 2C) showed a trend towards reduced frequency among offspring of supplemented mice (Fig. 2E), although this did not reach statistical significance. Treatment with thymidine alone, or in combination with either adenine or GMP, produced the lowest exencephaly frequencies, suggesting that thymidine might be the principal factor encouraging completion of cranial neurulation.

We next investigated the mechanism underlying the protective effect of supplemental nucleotides. Litter size and resorption rates were unaffected by treatment, indicating that reduction in neural tube defect frequency did not result from lethality of affected embryos (Table 1). To evaluate uptake of nucleotide precursors, mouse embryos were cultured from Days 9.5–10.5 in the presence of ^3^H-thymidine, ^3^H-adenine or ^3^H-hypoxanthine. Detection of ^3^H-labelling in genomic DNA (Supplementary Table 1) showed that supplemental nucleotides cross the yolk sac and are incorporated into DNA in the embryo. These data also confirm that the salvage enzymes TK, APRT and HPRT (Fig. 1) are active at neurulation stages.

**Table 1:** Litter size and resorption rate is not affected by nucleotide supplementation

Treatment	No. litters	Litter size (live embryos)	Resorptions per litter
**Control**	15	6.9 ± 0.5	0.6 ± 0.3
**Thy + Ade**	14	7.1 ± 0.4	0.2 ± 0.1
**Thy + Hyp**	8	8.0 ± 0.7	0.1 ± 0.1
**Thy + GMP**	9	8.9 ± 0.6	0.3 ± 0.2
**Ade only**	8	7.5 ± 0.7	0.8 ± 0.3
**Thy only**	10	7.4 ± 0.8	0.9 ± 0.4

The number of live embryos in each litter was recorded, together with the presence of any dead embryos or resorptions. Data are expressed as mean ± SEM. There was no statistically significant difference between treatments in the litter size or the number of dead/resorbed embryos per litter (one-way ANOVA). Thy = thymidine; Ade = adenosine; Hyp = hypoxanthine.

Spinal neural tube defects in *curly tail* embryos result from a reduced rate of cellular proliferation in the hindgut. The consequent imbalance in growth of dorsal and ventral tissues causes increased ventral curvature of the caudal region of the embryo and mechanical inhibition of neural fold closure (Brook *et al.*, 1991; Van Straaten and Copp, 2001). Transgenic overexpression of *Grhl3* or treatment with *myo*-inositol corrects this proliferation defect and prevents neural tube defects (Greene and Copp, 1997; Cogram *et al.*, 2004; Gustavsson *et al.*, 2007). As supplemental nucleotides are used in DNA synthesis, we hypothesized that the mechanism underlying prevention of neural tube defects in *curly tail* embryos involves enhanced proliferation.

**Figure 3:**
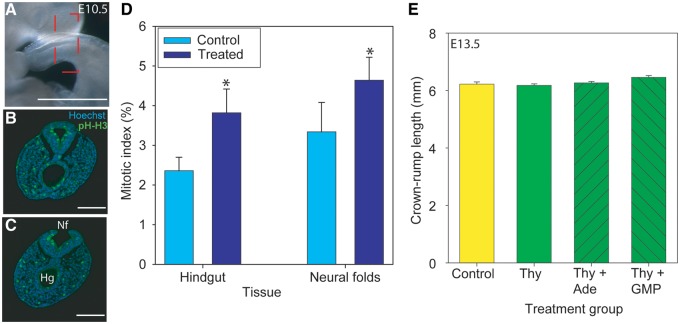
Supplemental nucleotides stimulate cellular proliferation. (**A–D**) At Day 10.5 (E10.5), during spinal neural tube closure, the mitotic index was determined at the level of the closure point of the neural folds at the posterior neuropore (boxed in **A**, which shows the caudal region of a representative treated embryo). Phosphohistone H3 (pH-H3)-positive cells were counted on transverse sections immediately anterior (**B**) and posterior (**C**) to the closure point. (**D**) Mitotic index was significantly elevated in the hindgut (Hg) and neural folds (Nf) of embryos treated with thymidine + adenine (asterisk indicates significant difference to controls; *P* < 0.05, *t*-test). Scale bars: **A** = 1 mm; **B–C** = 0.1 mm. *n* = seven treated and nine control embryos with seven to eight sections analysed per embryo. (**E**) Foetal size at Day 13.5 (E13.5), as determined by measurement of crown–rump length, did not significantly differ between control litters and those exposed to supplemental nucleotides at Days 7.5–10.5, suggesting that long-term growth is not affected after cessation of treatment. Data are expressed as mean ± SEM. Number of foetuses analysed: controls = 22; thymidine only (Thy) = 13; thymidine + adenine = 39; thymidine + GMP = 21. Ade = adenine.

We therefore analysed cell proliferation at Day 10.5, at the axial level of the closing spinal neural folds (Fig. 3A–C) and observed a significant increase in the mitotic index in the hindgut and neural folds of treated embryos compared with controls (Fig. 3D). Importantly, there was a 1.63-fold increase in hindgut mitotic index, but only a 1.39-fold increase in neuroepithelial mitotic activity. This proportionally greater effect of nucleotide supplementation on hindgut proliferation is expected to minimize the dorsal–ventral growth imbalance that is known to hamper neural fold closure in *curly tail* embryos. Moreover, the increase in mitotic index that we observed in the hindgut is similar to the 1.7-fold increase observed in *curly tail* embryos whose spina bifida was rescued by a *Grhl3* transgene (Gustavsson *et al.*, 2007). Taken together, these findings suggest that enhanced proliferation underlies the protective effect of supplemental nucleotides. Measurement of embryo size at Day 13.5, following cessation of treatment at Day 10.5, did not reveal a long-term effect of treatment on overall growth (Fig. 3E).

## Discussion

Defective thymidylate biosynthesis has been implicated in neural tube defects in humans and mouse models (Fleming and Copp, 1998; Dunlevy *et al.*, 2007; Beaudin *et al.*, 2011), suggesting that adequate supply of nucleotides for cellular proliferation is essential for neural tube closure. Supplementation with thymidine alone had minimal effect on spina bifida in the current study or in other mouse models (Wlodarczyk *et al.*, 2006; Burren *et al.*, 2008). In contrast, the significant protective effect of concurrent administration of adenine or GMP with thymidine suggests that provision of both pyrimidine and purine precursors effectively stimulates proliferation.

Folic acid provides a key tool in the prevention of neural tube defects. However, to mediate bioactivity supplemental folic acid must be metabolized through multiple reactions, defects in which could explain lack of response in some individuals. Downstream intervention using nucleotide precursors could potentially circumvent a scenario in which folic acid cannot ameliorate some FOCM-related neural tube defects owing to defects in the intervening enzymes required to metabolize folic acid. It is unclear whether the lack of protective effect of folic acid in the *curly tail* model reflects an underlying defect in FOCM. Nevertheless, *curly tail* embryos are sensitive to folate status, developing cranial neural tube defects at increased frequency under conditions of dietary folate deficiency (Burren *et al.*, 2010). Moreover, in comparison with wild-types, *curly tail* embryos display abnormalities in levels of methylation cycle intermediates, including increased abundance of *S*-adenosylhomocysteine (de Castro *et al.*, 2010). Impaired methylation is unlikely to contribute to neural tube defects in *curly tail* embryos, as the frequency of defects is not exacerbated in *ct/ct*; *Mthfr^−/−^* double mutant embryos (de Castro *et al.*, 2010). Nevertheless, altered levels of methylation cycle intermediates may reflect perturbation of FOCM.

Birth defects are the leading cause of infant mortality in developed nations and cause long-term health problems in surviving children. Among these conditions neural tube defects are so far unique in being amenable to primary prevention. However, the existence of a subset of neural tube defects that are resistant to folic acid means that further reduction in the rate of neural tube defects is likely to require development of adjunct therapies to be used in combination with folic acid. Direct supplementation with nucleotide precursors represents an avenue for further investigation and in humans has been found to be possible by oral means, for example in infant formula (Singhal *et al.*, 2010). We hypothesize that maternal supplementation with nucleotide precursors could not only prevent some folic acid-resistant neural tube defects but potentially enhance the protection afforded by folic acid in responsive cases.

## Funding

This work was supported by the Medical Research Council (J003794), Newlife Foundation (11-1206) and the Wellcome Trust (087525).

## Supplementary material

Supplementary material is available at *Brain* online.

Supplementary Data

## Abbreviations

FOCM: folate one-carbon metabolism
GMP: guanosine monophosphate
