# Supplementary material for: Nucleotide precursors prevent folic acid-resistant neural tube defects in the mouse
Source: Brain. 2013 Aug 9;136(9):2836–41. doi: 10.1093/brain/awt209 (PMC3754462; doi:10.1093/brain/awt209)
Supplement: Supplementary Data [file supp_136_9_2836__index.html]

Nucleotide precursors prevent folic acid-resistant neural tube defects in the mouse — Supplementary Data 

# Nucleotide precursors prevent folic acid-resistant neural tube defects in the mouse

## Supplementary Data

files

**Files in this Data Supplement:**

- Supplementary Data - docx file
