# Supplementary material for: Nucleotide precursors prevent folic acid-resistant neural tube defects in the mouse
Source: Brain. 2013 Aug 9;136(9):2836–41. doi: 10.1093/brain/awt209 (PMC3754462; doi:10.1093/brain/awt209)
Supplement: Supplementary Data [file supp_awt209_brain-2013-00224-File002.docx]

**Supplementary material**

| Treatment | Radioactivity (Bq/ml) | Amount of supplemental precursor (nmol/ml) | [^3^H] incorporation into genomic DNA (cpm/μg) |
| --- | --- | --- | --- |
| Controls | 0 | 0 | 6.3 ± 1.0 |
| [^3^H]-thymidine | 37,000 | 0.07 | 898.4 ± 98.0 |
| [^3^H]-hypoxanthine | 37,000 | 0.07 | 116.6 ± 9.3 |
| [^3^H]-adenine | 37,000 | 0.036 | 40.0 ± 6.6 |
| [^3^H]-adenine | 74,000 | 0.07 | 82.8 ± 6.7 |

**Table S1. Incorporation of exogenous nucleotide precursors into genomic DNA.** Embryos were cultured from E9.5-10.5 in the presence of tritiated compounds (n = 3 per treatment; doses indicated in Bq are equivalent to 1 or 2 µCi/ml). Labelling was normalised to the DNA content of the embryo and expressed as mean cpm/µg (± SEM). Based on measured cpm of nucleotide stock solutions an approximation of incorporation of [^3^H]-labelled molecule was calculated (for the 0.07 nmol/ml dose): **thymidine, 18.7 fmol/µg DNA; hypoxanthine, 2.9 fmol/µg DNA; adenine 1.2 fmol/µg DNA.** These data suggest that a greater amount of thymidine was incorporated into genomic DNA than adenine or hypoxanthine. However, the basal concentration of unlabelled molecules in the culture medium may have differed between nucleotides such that specific activity varied.

**
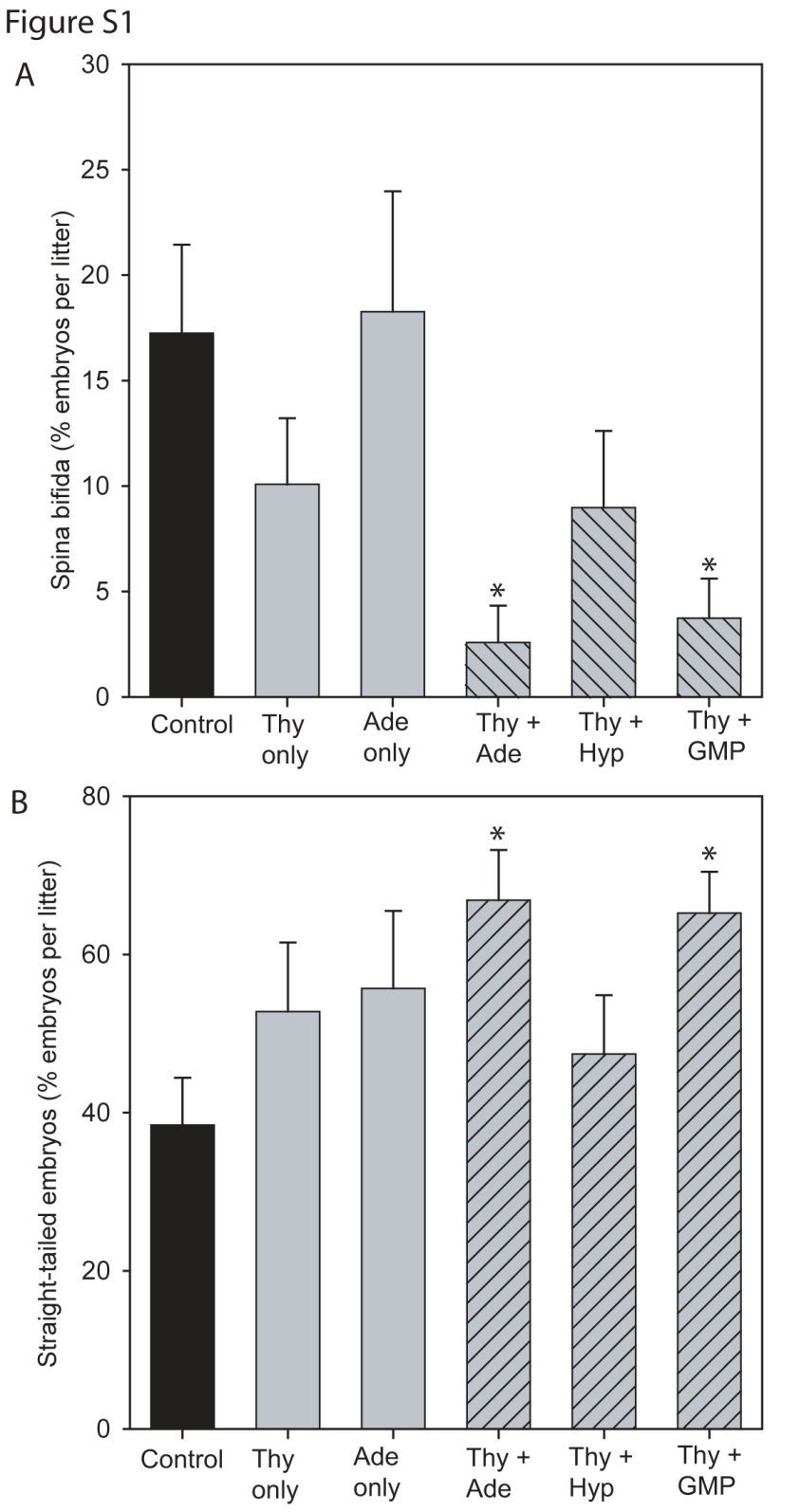
**

**Figure S1. Frequency of spina bifida and straight tails among litters of *curly tail* embryos supplemented with nucleotide precursors.** Offspring of mice treated with vehicle only (black bars), a single compound (grey bars) or combinations of compounds (hatched bars) were analysed. Among litters of mice treated with Thy + Ade or Thy + GMP, the proportion of (A) embryos affected by spina bifida was significantly lower than among control litters, while (B) in the same treatment groups the proportion of unaffected, straight tailed embryos, was significantly higher than among controls (*p<0.05; One Way ANOVA). Data is shown as mean % embryos within litter ± SEM.
